# Supplementary material for: Heightened immigration enforcement impacts US citizens’ birth outcomes: Evidence from early ICE interventions in North Carolina
Source: PLoS One. 2021 Feb 3;16(2):e0245020. doi: 10.1371/journal.pone.0245020 (PMC7857575; doi:10.1371/journal.pone.0245020)
Supplement: S1 Table — (DOCX) [file pone.0245020.s001.docx]

**Supplemental Information**

**S1 Table. List of counties included in the analysis.**

|  |  | **Newborns in the sample of analysis** | |
| --- | --- | --- | --- |
|  | County | *N* | % |
|  | Mecklenburg | 15,172 | 13.0 |
| *Future 287(g) adopters* | Alamance | 2,059 | 1.8 |
|  | Cabarrus | 2,749 | 2.3 |
|  | Cumberland | 5,177 | 4.4 |
|  | Durham | 4,195 | 3.6 |
|  | Gaston | 3,072 | 2.6 |
|  | Guilford | 6,411 | 5.5 |
|  | Henderson | 1,340 | 1.1 |
|  | Wake | 14,163 | 12.1 |
| *Future 287(g) applications denied* | Brunswick | 1,110 | 0.9 |
|  | Buncombe | 3,032 | 2.6 |
|  | Columbus | 745 | 0.6 |
|  | Duplin | 884 | 0.8 |
|  | Iredell | 2,368 | 2.0 |
|  | Lee | 941 | 0.8 |
|  | Randolph | 2,025 | 1.7 |
|  | Surry | 1,001 | 0.9 |
|  | Union | 3,053 | 2.6 |
|  | Yadkin | 534 | 0.5 |
| *Nonadopters and nonapplicants* | Beaufort | 2,858 | 2.4 |
|  | Bladen | 471 | 0.4 |
|  | Burke | 1,175 | 1.0 |
|  | Catawba | 2,227 | 1.9 |
|  | Chatham | 827 | 0.7 |
|  | Craven | 1,718 | 1.5 |
|  | Davidson | 2,271 | 1.9 |
|  | Forsyth | 4,816 | 4.1 |
|  | Franklin | 799 | 0.7 |
|  | Harnett | 1,559 | 1.3 |
|  | Hoke | 834 | 0.7 |
|  | Johnston | 2,672 | 2.3 |
|  | Lenoir | 889 | 0.8 |
|  | Montgomery | 430 | 0.4 |
|  | Moore | 1,073 | 0.9 |
|  | Nash | 1,349 | 1.2 |
|  | New Hanover | 2,664 | 2.3 |
|  | Onslow | 3,526 | 3.0 |
|  | Orange | 1,455 | 1.2 |
|  | Pitt | 2,257 | 1.9 |
|  | Robeson | 2,088 | 1.8 |
|  | Rockingham | 1,206 | 1.0 |
|  | Rowan | 1,976 | 1.7 |
|  | Sampson | 951 | 0.8 |
|  | Stanly | 833 | 0.7 |
|  | Wayne | 1,923 | 1.6 |
|  | Wilkes | 977 | 0.8 |
|  | Wilson | 1,148 | 1.0 |
|  | Total | 117,003 | 100.0 |
