# Supplementary material for: Heightened immigration enforcement impacts US citizens’ birth outcomes: Evidence from early ICE interventions in North Carolina
Source: PLoS One. 2021 Feb 3;16(2):e0245020. doi: 10.1371/journal.pone.0245020 (PMC7857575; doi:10.1371/journal.pone.0245020)
Supplement: S2 Table — (DOCX) [file pone.0245020.s002.docx]

**S2 Table. Change in birth outcomes and health care utilization over time within county of residence, by mother’s nativity and education.**

|  | **Difference-in-Differences Estimates [95% CI]^a^** | | |
| --- | --- | --- | --- |
|  | **Births to less-educated foreign-born mothers (N=17,884)^b^** | **Births to more-educated foreign-born mothers (N=16,366)** | **Births to non-Hispanic US-born mothers (N=117,885)** |
|  | **Adjusted FE-DEM^c^** | **Adjusted FE-DEM** | **Adjusted FE-DEM** |
| Birth weight, g | −36.33 | 13.2 | 8.58 |
|  | [−56.46, −16.19] | [−4.29, 30.68] | [0.95, 16.20] |
| Low birth weight | −0.27 | −0.30 | −0.06 |
|  | [−1.05, 0.51] | [−0.90, 0.29] | [−0.39, 0.28] |
| Small for gestational age | 1.63 | 1.14 | −0.32 |
|  | [0.61, 2.64] | [−0.15, 2.44] | [−0.68, 0.05] |
| Inadequate prenatal care | 9.22 | 4.26 | 0.72 |
|  | [7.08, 11.36] | [3.02, 5.51] | [0.37, 1.07] |

Abbreviations: FE, fixed effects; DEM, demographics.

^a^ The 95% confidence intervals within brackets are based on standard errors clustered at the county level.

^b^ Less-educated mothers have less than high school (or nonreported) education.

^c^ Adjusted FE-DEM model included county fixed effects, month-year fixed effects, and demographic controls (listed in Table 1).
